# Supplementary material for: Are All Societies Ready for Digital Tools? Feasibility Study on the Use of Mobile Application in Polish Early Breast Cancer Patients Treated with Perioperative Chemotherapy
Source: Healthcare (Basel). 2023 Jul 24;11(14):2114. doi: 10.3390/healthcare11142114 (PMC10379019; doi:10.3390/healthcare11142114)
Supplement: Supplementary file 1 [file healthcare-11-02114-s001.zip › healthcare-2446739-supplementary.pdf]

Table S1. Detailed symptom assessment scale

	0	1	2	3	4
Pain					
	I don't feel pain.	I feel mild pain, that doesn't affect my usual daily activities.	I feel moderate pain, that affects my usual daily activities, but I'm still able to cope.	I feel severe pain, that seriously affects my basic daily activities.	I feel completely exhausted by pain.
Fatigue					
	I don't feel fatigued, comparing to my usual state.	I feel mild fatigue, that is relieved by rest.	I feel moderate fatigue, that is not relieved by rest AND/OR is causing moderate problems with my usual daily activities.	I feel severe fatigue, that seriously affects my basic daily activities.	I feel completely exhausted.<br>I can't cope.
Nausea					
	I don't feel nausea.	I lost appetite because of nausea but I'm able to eat as usual.	I eat and drink less than usual because of nausea, but my weight is stable and<br>I'm not dehydrated or malnourished	I don't eat and drink enough OR I'm dehydrated or malnourished, OR nausea seriously affects my basic daily activities.	________
Vomiting					
	I wasn't vomiting	I was vomiting, but less than 2 times per 24 hours	I was vomiting 3-5 times per 24 hours	I was vomiting more than 6 times per 24 hours OR I'm dehydrated and/or malnourished, OR vomiting seriously affects my basic daily activities.	I feel completely exhausted by vomiting.
Diarrhoea					
	I don't have more bowel movements than usual	I have 1-3 more bowel movements than usual	Every day, I have 4-6 more bowel movements than usual, but I'm not dehydrated and diarrhoea doesn't affect my basic daily activities.	I have more than 6 bowel movements per 24 hours OR diarrhoea seriously affects my basic daily activities.	I feel completely exhausted by diarrhoea.
Constipation					

	I don’t have constipation.	I have occasional or intermittent constipation OR use stool softeners, laxatives, dietary modification or enema occasionally.	I have persistent constipation and use laxatives or enemas regularly.	Constipation seriously affects my basic daily activities OR manual evacuation of stool was necessary.	I feel completely exhausted by constipation.
Appetite loss					
	I don’t have problems with my appetite or eating.	I lost appetite but I eat and drink as usual.	I eat less but my weight is stable and I’m not malnourished.	I eat or drink very little and I lose weight, AND/OR I’m malnourished.	I feel completely exhausted by malnutrition caused by appetite loss.
Oral mucositis					
	I don’t have oral mucositis.	I have mild mucositis but it doesn’t cause any discomfort and I’m able to eat as usual.	I have moderate mucositis, that is causing some discomfort but I’m able to eat soft diet.	I have severe mucositis, I’m not able to eat or drink.	I feel completely exhausted by mucositis
Pins and needles in hands and feet					
	I don’t feel pins and needles in my hands and feet.	I feel mild pins and needles in my hands and feet, but it doesn’t affect my daily activities like fastening buttons, feeling for small object or walking	I feel moderate pins and needles in my hands and feet OR pins and needles in hands and feet challenge my daily activities like fastening buttons, feeling for small object or walking	I feel severe pins and needles in my hands and feet OR I’m not able to do daily activities like fastening buttons, feeling for small object or walking, because of pins and needles in my hands and feet.	I spent most of the day in bed because of severe pins and needles in my hands and feet.
Cough					
	I don’t have cough.	I have mild cough OR cough is relieved by over the counter medication, herbs or supplements.	I have persistent cough, that limits some daily activities OR I have to take prescription medication to relieve cough.	I have constant cough OR I have problems with basic daily activities like eating, washing, walking or sleeping.	———
Shortness of breath					
	I don’t have shortness of breath.	I have mild shortness of breath with moderate exertion but I	I have moderate shortness of breath with mild exertion. I can’t walk to the	I have severe shortness of breath even when resting OR it is very difficult for me to	I can’t breathe. I’m not able to do anything.

		can still walk to the first floor or walk around the house without resting.	first floor or walk around the house without resting.	do daily activities like eating, washing, walking or sleeping, because of severe shortness of breath.	
Hand and foot syndrome					
	I don’t observe erythema, peeling or blistering on my hands or feet.	I have erythema on my hands and feet but it doesn’t affect my usual daily activities, working or walking.	I have peeling, bleeding, blistering or oedema of hands and feet, AND/OR I have difficulties with my usual daily activities, working, walking because of pain in my hands and feet.	I have serious difficulties with basic daily activities because of skin changes and pain.	________
Fever					
	I don’t have fever.	I have fever between 38.1-39^{0}C.	I have fever between 39.1-40^{0}C	I have 40^{0}C fever for less than 24 hours.	I have 40^{0}C fever for more than 24 hours.
Dizziness					
	I don’t feel dizzy.	I feel mildly unstable, mildly off balance when walking.	I feel moderately unstable. It limits my usual daily activities.	I feel seriously unstable and dizzy. I’m not able to do daily activities independently.	________

Table S2. Symptoms reported in relation to categorical sociodemographic and medical factors.

	Fatigue			Dizziness			Gastrointestinal problems			Respiratory problems			Other			All		
	n/mean	median	p	n/mean	median	p	n/mean	median	p	n/mean	median	p	n/mean	median	p	n/mean	median	p
**Place of residence**																		
Rural area	170/8	7	0.89	88/4	1	0.58	214/10	7.5	0.85	113/5	2	0.24	259/12	7.5	0.40	844/38	28.5	0.17
City < 50 000	47/6	5		13/2	1		43/5	5		15/2	0.5		33/4	3		151/19	19	
City > 50 000	211/8	5		95/4	1		235/9	7		81/3	1		191/8	4		813/32.5	22	
**Education**																		
Vocational	62/7	8	0.81	21/2	3	0.88	67/7	6	0.34	22/2	2	0.26	60/7	4	0.84	232/26	28	0.12
Secondary	80/7	4		42/4	1		91/8	2		39/3.5	0		111/10	2		363/33	7	
Higher	286/8	5		133/4	1		334/9.5	8		148/4	3		312/9	5		1213/35	28	
**Employment status**																		
Unemployed	32/16	16	0.22	4/2	2	0.80	18/9	9	0.51	26/13	13	0.04*	35/17.5	17.5	0.39	115/57.5	57.5	0.02*
Employed	295/7	5		149/3.5	1		343/8	5.5		110/3	1		320/8	3.5		1217/29	22	
Retired	101/9	5		43/4	1		131/12	10		73/7	4		128/12	4		476/43	30	
**Marital status**																		
Single	79/9	5	0.69	41/5	1	0.34	109/12	8	0.20	52/6	2	0.10	126/14	9	0.24	407/45	22	0.002*
In relationship	27/9	8		14/5	4		36/12	14		22/7	4		31/10	13		130/43	55	
Married	291/7	5		131/3	1		335/9	6		135/3	1		299/8	4		1191/30.5	23	
Divorced	18/6	1		3/1	1		5/2	0		0	0		10/3	0		36/12	2	
Widow	13/13	13		7/7	7		7/7	7		0	0		17/14	17		44/44	44	
**Patient's economic status (self-assessed)**																		
Unsatisfactory	100/10	9	0.27	47/5	2	0.40	102/10	6.5	0.75	46/5	2	0.48	127/13	6	0.40	422/42	28.5	0.12
Satisfactory	328/7	5		149/3	1		390/9	7		163/4	1		356/8	4		1386/31	22	
**Breast cancer stage**																		

I	102/8.5	3	0.40	55/5	1	0.62	88/7	4	0.40	10/1	0	0.08	76/6	2.5	0.60	331/28	14	0.03*
II	196/7	5		88/3	2		250/9	7		130/4	3		246/8	4		910/31	28	
III	130/9	8		53/4	1		154/11	7.5		69/5	2		161/11.5	6.5		567/40.5	29.5	
**Coexisting medical conditions**																		
No	189/9	5	1	83/4	1	0.40	150/7	5	0.11	70/3	1	0.51	162/8	2	0.50	654/31	16	0.13
Yes	239/7	5		113/3	1.5		342/10	8		139/4	2		321/9	5		1154/34	26	
**Chemotherapy setting**																		
Adjuvant	95/6	3	0.55	41/3	2	0.85	103/7	5	0.34	61/4	1	0.50	125/8	3	0.96	425/28	16	0.40
Neoadjuvant	333/8	5		155/4	1		389/10	7		148/4	1.5		358/9	4		1383/35	28	
**Number of chemotherapy administrations**																		
4	22/4	2	0.59	16/3	1	0.76	24/5	3	0.63	18/4	0	0.87	34/7	1	0.72	114/23	6	0.35
6	4/4	4		3/3	3		6/6	6		1/1	1		2/2	2		16/16	16	
12	36/9	5		5/1	0.5		22/5.5	4.5		23/6	0.5		37/9	4		123/31	15.5	
16	366/8	6		172/4	1		440/10	7		167/4	2		410/9	5		1555/35	28	
**Treatment with G-CSF**																		
No	234/8	6	0.41	91/3	1	0.82	246/8	6	0.40	123/4	1	0.76	283/10	5	0.73	977/34	24	0.97
Yes	194/7	5		105/4	1		246/9	7.5		86/3	1.5		200/8	3.5		831/32	22.5	
**Chemotherapy regimen**																		
Taxane or anthracycline	58/6	3	0.49	24/3	1	0.83	51/6	5	0.37	42/5	1	0.65	71/8	2	0.61	246/27	16	0.29
Taxane + anthracycline	370/8	5.5		172/4	1		441/10	7		167/4	1.5		412/9	4.5		1562/34	28	

* - values not confirmed by Wilcoxon signed-rank test; G-CSF- granulocyte colony stimulating factors
